# Supplementary material for: Advances in the Biosynthesis and Molecular Evolution of Steroidal Saponins in Plants
Source: Int J Mol Sci. 2023 Jan 30;24(3):2620. doi: 10.3390/ijms24032620 (PMC9917158; doi:10.3390/ijms24032620)
Supplement: Supplementary file 1 [file ijms-24-02620-s001.zip › ijms-2150483-supplementary.pdf]

Supplementary Table S1. Steroidal saponins isolated from plant.

Steroidal saponins	Species name	Plant family	Tissue/Organ	Reference
Agavoside A/C/I, Chlorogenin 3,6-di-O-b-D-glucopyranoside, Hainangenin 3,6-di-O-b-D-glucopyranoside, Hongguanggenin 3,6-di-O-b-D-glucopyranoside, Agamenoside B/H	*Agave americana*	Agavaceae	Leaves	[1]
One monosaccharide, two tetrasaccharides, four pentasaccharides, and three hexasaccharide spirostanol saponins	*Agave cantala*	Agavaceae	Fruits	[2]
Tigogenin 3-O-b-D-glucopyranoside, Sarsasapogenin 3-O-b-D-glucopyranoside	*Agave kerchovei*	Agavaceae	Whole plant	[3]
Four tetrasaccharide and six pentose spirosteroid saponins, five triglycosyl furostanol saponins	*Agave offoyana*	Agavaceae	Leaves, flowers	[4]
Three disaccharide and three trisaccharide furostanol saponins	*Agave sisalana*	Agavaceae	Leaves	[4]
Chlorogenin 3-O-b-D-glucopyranoside, Chlorogenin 6-O-b-D-glucopyranoside, ten trisaccharide and four tetrasaccharide spirostanol saponins	*Agave utahensis*	Agavaceae	Whole plant	[5]
Three spirostanol saponins and two furostanol saponins	*Camassia leichtlinii*	Agavaceae	Corms	[1]
Three spirostanol saponins, one furostanol saponin and one cholestanol saponin	*Yucca gloriosa*	Agavaceae	Flowers	[6]

Supplementary Table S1. continued.

Steroidal saponins	Species name	Plant family	Tissue/Organ	Reference
Three furostanol saponins, one spirostanol saponin, and five steroidal saponins specific to *Yucca*	*Yucca schidigera*	Agavaceae	Bark	[7]
One cholestane saponin	*Allium albopilosum*	Allioideae	Seeds	[8]
Two furostanol spaonins and agigenin saponin	*Allium atroviolaceum*	Allioideae	Bulbs	[9]
Ceparosides C and D	*Allium cepa*	Allioideae	Seeds	[10]
Fistulosaponins A–F	*Allium fistulosum*	Allioideae	Seeds	[11]
One spirostanol saponin, two agigenin saponins	*Allium flavum*	Allioideae	Seeds	[11]
Five spirostanol saponins (two β-Chlorogenin as the aglycone, three agigenin as the aglycone)	*Allium leucanthum*	Allioideae	Flowers	[12]
Three spirostanol saponins and twenty-four furostanol saponins	*Allium macrostemon*	Allioideae	Carmus	[9]
Three cholestane type steroidal saponins	*Allium schubertii*	Allioideae	Seeds	[8]
Sixteen spirostanol saponins and one cholestane type steroidal saponin	*Allium tuberosum*	Allioideae	Whole plant	[8,13]
Two spirostanol saponins (Diosgenin as the aglycone)	*Allium ursinum*	Allioideae	Underground parts	[13]
Protodioscin, 3-o-histi-dine-26-o-hexosyl-dioscin, 3-o-histidine-26-o-dihexosyl hydroxydioscin	*Phoenix dactylifera*	Arecaceae	Pollens	[14]
Filiasparosides A–G, Aspafiliosides A/B/E/F and Asparagusin A	*Asparagus filicinus*	Asparagoideae	Roots	[15,16]
Protodioscin, Sarsasapogenin M/N and Yamogenin II	*Asparagus officinalis*	Asparagoideae	Roots	[17]

Supplementary Table S1. continued.

Steroidal saponins	Species name	Plant family	Tissue/Organ	Reference
Racemosides A-C, Shatavarins VI–X, Shatavaroside A/ B/C and Filiasparoside C	*Asparagus racemosus*	Asparagoideae	Roots	[18]
24(S)-Hydroxy-neotokorogenin and Isorhodea sapogenin	*Hemerocallis fulva var. kwanso*	Asphodelaceae	Aerial parts	[19]
Dioscin, Gracillin, Protogracillin, Parvifloside, Costusoside I/J	*Costus speciosus*	Costaceae	Rhizomes	[20]
Furostanol saponins	*Costus spiralis*	Costaceae	Rhizomes	[21]
Dioscoreanosides A–K	*Dioscorea bulbifera*	Dioscoreaceae	Tubers	[22]
Parvifloside, Methyl protogracillin, Protodioscin and Methyl protodioscin	*Dioscorea collettii* var. *hypoglauca*	Dioscoreaceae	Rhizomes	[23]
Parvifloside, Methyl protogracillin, Protodioscin and Methyl protodioscin	*Dioscorea deltoidea*	Dioscoreaceae	Tubers	[24]
Four furostanol saponins and eleven spirostanol saponins	*Dioscorea esculenta*	Dioscoreaceae	Tubers	[25]
Pseudoprotodioscin, protodiosgenin, methyl protodioscin, protoneodioscin, protoslender diosgenin, Gracillin	*Dioscorea futschauensis*	Dioscoreaceae	Rhizomes	[24]
Pseudoprotodioscin, Methyl protodioscin and Dioscin	*Dioscorea nipponica*	Dioscoreaceae	Rhizomes	[26]

Supplementary Table S1. continued.

Steroidal saponins	Species name	Plant family	Tissue/Organ	Reference
Dioscin	*Dioscorea panthaica*	Dioscoreaceae	Tubers	[24,27,28]
Dioscin, Deltonin, Parvifloside, Methyl parvifloside, Methyl deltoside	*Dioscorea parviflora*	Dioscoreaceae	Rhizomes	[24]
Diospolysaponin A and three spirostanol saponins	*Dioscorea polygonoides*	Dioscoreaceae	Tubers	[25]
Diospreussinosides A–C	*Dioscorea preussii*	Dioscoreaceae	Rhizomes	[29]
Spongipregnoloside A-D, Spongioside A/B,Trigofoenoside D-1 and Hypoglaucin G	*Dioscorea septemloba*	Dioscoreaceae	Rhizomes	[28]
Spongipregnoloside A/B/C/D; Spongioside A/B;	*Dioscorea spongiosa*	Dioscoreaceae	Rhizomes	[24,30]
Protodioscin	*Dioscorea tokoro*	Dioscoreaceae	Rhizomes	[24]
Parvifloside, Methyl protodeltonin, Zingiberensis saponin I, Deltonin and Dioscin	*Dioscorea villosa*	Dioscoreaceae	Rhizomes	[24]
Seventy steroidal saponins including Dioscin, Gracillin, Protogracillin, Parvifloside, Protodeltonin, Zingiberensis saponin,Trillin, Methyl protodioscin, Methyl protogracillin, Protobioside, Pseudoprotodioscin, Pseudoprotogracillin	*Dioscorea zingiberensis*	Dioscoreaceae	Rhizomes	[31,32]
Six furostanol saponins	*Trigonella foenum graecum*	Fabaceae	seeds	[33,34]

Supplementary Table S1. continued.

Steroidal saponins	Species name	Plant family	Tissue/Organ	Reference
Pallidiflosides D/E/G/ H/I, Spongipregnoloside A, Smilaxchinoside C, Timosaponin H1, Protobioside, Polygonatoside B3, Polyphyllin V and Deltonin	*Fritillaria pallidiflora*	Liliaceae	Bulbs	[35]
Six steroidal saponins	*Lilium callosum*	Liliaceae	Bulbs	[36]
Eight spirostanol saponins	*Lilium candidum*	Liliaceae	Bulbs	[37]
Seven spirostanol saponins and three furostanol saponins	*Lilium longiflorum*	Liliaceae	Bulbs	[36]
Six steroidal saponins	*Lilium pumilum*	Liliaceae	Bulbs	[38]
Two steroidal saponins	*Lilium speciosum*	Liliaceae	Bulbs	[38]
Tenuifolioside A and B (polyhydroxylated steroidal saponin)	*Lilium tenuifolium*	Liliaceae*m*	Bulbs	[38]
Two spirostanol saponins and one cholestane-type saponin	*Cordyline fruticosa*	Lomandroideae	Leaves	[39]
Three spirostanol saponins and two furostanol saponins	*Cordyline stricta*	Lomandroideae	Leaves	[40]
Eleven steroidal saponin	*Paris delavayi*	Melanthiaceae	Rhizomes	[41]
Trillin, Dioscin, Polyphyllin C/D/G	*Paris polyphylla*	Melanthiaceae	Rhizomes	[42]
Twenty-one Steroidal saponins, including nineteen spirostanol saponins	*Paris polyphylla* var. *chinensis*	Melanthiaceae	Rhizomes	[41]

Supplementary Table S1. continued.

Steroidal saponins	Species name	Plant family	Tissue/Organ	Reference
Ten steroidal saponins	*Trillium erectum*	*Melanthiaceae*	Roots	[43]
Eighteen steroidal saponins	*Trillium kamtschaticum*	*Melanthiaceae*	Roots	[43]
Six steroidal saponins	*Trillium tschonoskii*	*Melanthiaceae*	Roots	[43]
Disporoside A/B/C/D	*Disporopsis pernyi*	Nolinoideae	Rhizomes	[44]
Twenty-two spirostanol saponins, four furostanol saponins and two pregnane saponins, including Namonin A/B/C/D/E/F	*Dracaena angustifolia*	Nolinoideae	Roots and rhizomes	[45,46]
Cambodianoside A/B/C/D/E/F/G	*Dracaena cambodiana*	Nolinoideae	Stems	[45]
Dracaenoside C/D/E/G/H/I/J/K/L/M/N/O/P/Q/R	*Dracaena cochinchinensis*	Nolinoideae	Stems	[45]
Three pennogenin saponins and two β-Sitosterol saponins	*Dracaena draco*	Nolinoideae	Aerial parts; roots	[45]
(25R,22)-hydroxylwattinoside C, Kingianoside F, 22-hydroxylwattinoside C, Kingianoside C/D/E, (25S)-kingianoside C/D/E/F, Gracillin	*Polygonatum kingianum*	Nolinoideae	Rhizomes	[29]
Polygonatumoside A/B/C, Polyfuroside, Methyl protoaspidistrin, Polygodoside G, (25R)-Polygodoside E/F and (25R)-Polygodosin A	*Polygonatum odoratum*	Nolinoideae	Rhizomes	[47]

Supplementary Table S1. continued.

**Steroidal saponins**	**Species name**	**Plant family**	**Tissue/Organ**	**Reference**
Dioscin and Polypunctoside A/B/C/D	*Polygonatum punctatum*	Nolinoideae	Rhizomes	[47]
Two cholestane saponins, Sibiricoside A, Huangjinoside P and Polygonoide A	*Polygonatum sibiricum*	Nolinoideae	Rhizomes	[47]
Twenty-six spirostanol saponins, six furostanol saponins, four pregnane saponins	*Sansevieria trifasciata*	Nolinoideae	Aerial parts	[45]
Sixteen steroidal saponins, including Avenacoside A/B/C/D	*Avena sativa*	Poaceae	Leaves	[48]
Polyhydroxy hellebosaponins	*Helleborus niger*	Ranunculaceae	Rhizomes	[49,50]
Two spirostanol saponins and one furostanol saponin	*Helleborus thibetanus*	Ranunculaceae	Roots and rhizomes	[49,50]
Two spirostanol saponins	*Helleborus viridis*	Ranunculaceae	Leaves	[49,50]
Curillin G, Asparagoside E, Asparoside A/B	*Smilax aspera susp. mauritanica*	Smilacaceae	Roots	[51]
Five steroidal saponins	*Smilax bockii*	Smilacaceae	Rhizomes	[51]
Dioscin, Methylprotodioscin, Protodioscin, Pseudoprotodioscin, 15-Hydroxypseudoprotodioscin, Smilaxchinoside A/B/C/D	*Smilax china*	Smilacaceae	Roots and tubers	[39,51,52]
Three furosteroidal saponins linked to acetyl glycogroups	*Smilax excelsa*	Smilacaceae	Aerial parts	[39,51]

Supplementary Table S1. continued.

Steroidal saponins	Species name	Plant family	Tissue/Organ	Reference
Six smilagenin saponins	*Smilax medica*	Smilacaceae	Rhizomes	[39,51]
Three Sarsasapogenin glycosides	*Smilax ornata*	Smilacaceae	Roots	[53]
Parisvietnaside A, Parisyunnanoside A/C/D/E, Protogracillin, Parisaponin I, (25R)-Smilaxchinoside A, Smilaxchinoside C	*Smilax riparia*	Smilacaceae	Rhizomes, roots	[39,51]
Dioscin, Methylprotodioscin, Pseudoprotodioscin	*Smilax stans*	Smilacaceae	Roots	[39,51]
Four spirostanol saponins and two furostanol saponins	*Cestrum nocturnum*	Solanaceae	Leaves	[54]
Three furostanol saponins	*Solanum abutiloides*	Solanaceae	Rhizomes	[55]
Four spirostanol saponins and one furostanol saponin	*Solanum anguivi*	Solanaceae	Rhizomes	[56]
Five furostanol saponins, two cholestane saponins, one spirostanol saponin	*Solanum melongena*	Solanaceae	Rhizomes	[57]
Eleven steroidal saponins	*Solanum torvum*	Solanaceae	Rhizomes	[58]
Five furostanol saponins and two spirostanol saponins	*Tacca chantrieri*	Taccaceae	Rhizomes	[59]
Seventeen steroidal saponins	*Tribulus terrestris*	Tribuloideae	Rhizomes	[60]

References

1. Jin, J.-M.; Zhang, Y.-J.; Yang, C.-R. Four New Steroid Constituents from the Waste Residue of Fibre Separation from Agave americana Leaves. *Chem. Pharm. Bull.* **2004**, 52, 654–658.
2. Sati, O.P.; née Pant, G.J.; Miyahara, K.; Kawasaki, T. Cantalasaponin-1, a Novel Spirostanol Bisdesmoside from *Agave cantala*. *J. Nat. Prod*.**1985**, 48, 395–399.

3. El-hashash, M.; Amine, M.S.; Shoeb, H.A.; Refahy, L.A. Triterpenes from *Agave kerchovei*. ChemInform **2010**, 26.
4. Sidana, J.; Singh, B.; Sharma, O.P. Saponins of *Agave*: Chemistry and Bioactivity. *Phytochemistry* **2016**, 130, 22–46.
5. Yokosuka, A.; Mimaki, Y. Steroidal Saponins from the Whole Plants of *Agave* utahensis and Their Cytotoxic Activity. *Phytochemistry* **2009**, 70, 807–815.
6. Montoro, P.; Skhirtladze, A.; Perrone, A.; Benidze, M.; Kemertelidze, E.; Piacente, S. Determination of Steroidal Glycosides in *Yucca gloriosa* Flowers by LC/MS/MS. *J. Pharm. Biomed. Anal.* **2010**, 52, 791–795.
7. Miyakoshi, M.; Tamura, Y.; Masuda, H.; Mizutani, K.; Tanaka, O.; Ikeda, T. Antiyeast Steroidal Saponins from *Yucca schidigera* (Mohave Yucca), a New Anti-Food-Deteriorating Agent. *J. Nat. Prod.* **2000**, 25, 332–338.
8. Hu, G.; Mao, R.; Ma, Z. A New Steroidal Saponin from the Seeds of *Allium tuberosum*. *Food Chem.* **2009**, 113, 1066–1068.
9. Sobolewska, D.; Michalska, K.; Podolak, I.; Grabowska, K. Steroidal Saponins from the Genus *Allium*. *Phytochem. Rev.* **2016**, 15, 1–35.
10. Yuan, L.; Ji, T.-F.; Li, C.-J.; Wang, A.-G.; Yang, J.-B.; Su, Y.-L. Two New Steroidal Saponins from the Seeds of *Allium cepa* L. J.f *Asian Nat. Prod. Res.* **2009**, 11, 213–218.
11. Lai, W.; Bo, Y.; Li, X.; Na, L.; Jun, Z.; Sheng, W. New Steroidal Sapogenins from the Acid Hydrolysis Product of the Whole Glycoside Mixture of Welsh Onion Seeds. *Chinese Chem. Lett.* **2012**, 23, 193–196.
12. Elias, R.; Pichette, A. Cytotoxic Steroidal Saponins from the Flowers of *Allium leucanthum*. *Molecules* **2008**, 2925–2934.
13. Jabrane, A.; Ben, H.; Miyamoto, T.; Mirjolet, J.; Duchamp, O.; Harzallah-skhiri, F.; Lacaille-dubois, M. Spirostane and Cholestane Glycosides from the Bulbs of *Allium nigrum* L. *Food Chem.* **2011**, 125, 447–455.
14. Hamed, A.I.; Ben Said, R.; Al-Ayed, A.S.; Moldoch, J.; Mahalel, U.A.; Mahmoud, A.M.; Elgebaly, H.A.; Perez, A.J.; Stochmal, A. Fingerprinting of Strong Spermatogenesis Steroidal Saponins in Male Flowers of *Phoenix dactylifera* (Date Palm) by LC-ESI-MS. *Nat. Prod. Res.* **2017**, 31, 2024–2031.
15. Zhou, L.-B.; Chen, T.-H.; Bastow, K.F.; Shibano, M.; Lee, K.-H.; Chen, D.-F. Filiasparosides A-D, Cytotoxic Steroidal Saponins from the Roots of *Asparagus filicinus*. *J. Nat. Prod.* **2007**, 70, 1263–1267.
16. Wu, J.-J.; Cheng, K.-W.; Zuo, X.-F.; Wang, M.-F.; Li, P.; Zhang, L.-Y.; Wang, H.; Ye, W.-C. Steroidal Saponins and Ecdysterone from *Asparagus filicinus* and Their Cytotoxic Activities. *Steroids* **2010,** 75, 734–739.
17. Huang, X.; Kong, L. Steroidal Saponins from Roots of *Asparagus officinalis*. *Steroids* **2006**, 71, 171–176.
18. Sharma, U.; Kumar, N.; Singh, B. Furostanol Saponin and Diphenylpentendiol from the Roots of *Asparagus racemosus*. *Nat. Prod. Commun.* **2012**, 7, 995–998.
19. Onishi, T.K.; Ujiwara, Y.F.; Onoshima, T.K.; Iyosawa, S.K.; Ishi, M.N. Steroidal Saponins from *Hemerocallis fulva* var . kwanso. *Chem. Pharm. Bull.* **2001**, 49, 318–320.

20. Pawar, V.; Pawar, P. *Costus speciosus*: An Important Medicinal Plant. *Ijsr.Net* **2014**, 3, 28–33.
21. Da Silva, B.P.; Parente, J.P. New Steroidal Saponins from Rhizomes of *Costus spiralis*. *Zeitschrift fur Naturforschung - Section C J. Biosci*. **2004**, 59, 81–85.
22. Ghosh, S. Phytochemsitry and Therapeutic Potential of Medicinal Plant: *Dioscorea bulbifera*. *Med. Chem.* **2015**, 5, 10259.
23. Yang, D.-J.; Lu, T.-J.; Hwang, L.S. Isolation and Identification of Steroidal Saponins in Taiwanese Yam Cultivar (*Dioscorea pseudojaponica* Yamamoto). *J. Agric. Food Chem.* **2003**, 51, 6438–6444.
24. Sautour, M.; Mitaine-Offer, A.C.; Lacaille-Dubois, M.A. The *Dioscorea* Genus: A Review of Bioactive Steroid Saponins. *J. Nat. Med.* **2007**, 61, 91–101.
25. Lee, H.J.; Watanabe, B.; Nakayasu, M.; Onjo, M.; Sugimoto, Y.; Mizutani, M. Novel Steroidal Saponins from *Dioscorea esculenta* (Togedokoro). *Biosci. Biotechnol. Biochem.* **2017**, 81, 2253–2260.
26. Lin, S.; Wang, D.; Yang, D.; Yao, J.; Tong, Y.; Chen, J. Characterization of Steroidal Saponins in Crude Extract from *Dioscorea nipponica* Makino by Liquid Chromatography Tandem Multi-Stage Mass Spectrometry. *Anal. Chim. Acta* **2007**, 599, 98–106.
27. Wang, W.; Zhao, Y.; Jing, W.; Zhang, J.; Xiao, H.; Zha, Q.; Liu, A. Ultrahigh-Performance Liquid Chromatography-Ion Trap Mass Spectrometry Characterization of the Steroidal Saponins of *Dioscorea panthaica* Prain et Burkill and Its Application for Accelerating the Isolation and Structural Elucidation of Steroidal Saponins. *Steroids* **2015**, 95, 51–65.
28. Yi, T.; Fan, L.L.; Chen, H.L.; Zhu, G.Y.; Suen, H.M.; Tang, Y.N.; Zhu, L.; Chu, C.; Zhao, Z.Z.; Chen, H.B. Comparative Analysis of Diosgenin in *Dioscorea* Species and Related Medicinal Plants by UPLC-DAD-MS. *BMC Biochem.* **2014**, 15, 1–6.
29. Acta, H.C.; Yu, H.; Song, X.; Kang, L.; Zhang, T.; Fu, J.; Zhao, Y.; Xiong, C.; Zhang, L.; Zhang, J.; et al. Two New Steroidal Saponins from the Processed *Polygonatum kingianum*. *Helv. Chim. Acta.* **2010**, 93, 1086–1092.
30. Yin, J.; Kouda, K.; Tezuka, Y.; Tran, Q. L.; Miyahara, T.; Chen, Y.; & Kadota, S. Steroidal glycosides from the rhizomes of *Dioscorea spongiosa*. *J. Nat. Prod.* **2003,** 66, 646–650.
31. Li, X.; Wang, Y.; Sun, J.; Li, X.; Zhao, C.; Zhao, P.; Man, S.; Gao, W. Chemotaxonomic Studies of 12 *Dioscorea* Species from China by UHPLC-QTOF-MS/MS Analysis. *Phytochem. Anal.* **2020**, 31, 164–182.
32. Zhang, X.; Jin, M.; Tadesse, N.; Dang, J.; Zhou, T.; Zhang, H.; Wang, S.; Guo, Z.; Ito, Y. *Dioscorea zingiberensis* C. H. Wright: An Overview on Its Traditional Use, Phytochemistry, Pharmacology, Clinical Applications, Quality Control, and Toxicity. *J. Ethnopharmacol.* **2018**, 220, 283–293.
33. Ciura, J.; Szeliga, M.; Grzesik, M.; Tyrka, M. Next-Generation Sequencing of Representational Difference Analysis Products for Identification of Genes Involved in Diosgenin Biosynthesis in Fenugreek (*Trigonella foenum-graecum*). *Planta* **2017**, 245, 977–991.

34. Murakami, T.; Kishi, A.; Matsuda, H.; Yoshikawa, M. Medicinal Foodstuffs. XVII. Fenugreek Seed. (3): Structures of New Furostanol-Type Steroid Saponins, Trigoneosides Xa, Xb, XIb, XIIa, XIIb, and XIIIa, from the Seeds of Egyptian *Trigonellafoenum-graecum* L. *Chem. Pharm. Bull.* **2000**, 48, 994–1000.
35. Shen, S.; Li, G.; Huang, J.; Chen, C.; Ren, B.; Lu, G.; Tan, Y.; Zhang, J.; Li, X.; Wang, J. Steroidal Saponins from *Fritillaria pallidiflora* Schrenk. *Fitoterapia* 2012, 83, 785–794.
36. Wang, P.; Li, J.; Attia, F.A.K.; Kang, W.; Wei, J.; Liu, Z.; Li, C. A Critical Review on Chemical Constituents and Pharmacological Effects of *Lilium*. *Food Sci. Hum. Wellness* **2019**, 8, 330–336.
37. Mimaki, Y.; Satou, T.; Kuroda, M.; Sashida, Y.; Hatakeyama, Y. New Steroidal Constituents from the Bulbs of *Lilium candidum*. *Chem. Pharm. Bull.* **1998**, 46, 1829–1832.
38. Mimaki, Y.; Sashida, Y. Steroidal and Phenolic Constituents of *Lilium speciosum*. *Phytochemistry* **1991**, 30, 937–940.
39. Challinor, V.L.; De Voss, J.J. Open-Chain Steroidal Glycosides, a Diverse Class of Plant Saponins. *Nat. Prod. Rep.* **2013**, 30, 429–454.
40. Mimaki, Y., Kuroda, M., Takaashi, Y., & Sashida, Y. Steroidal saponins from the leaves of *Cordyline stricta*. *Phytochemistry*, **1998**, 47, 79–85.
41. Wang, Y.; Gao, W.; Li, X.; Wei, J.; Jing, S.; Xiao, P. Chemotaxonomic Study of the Genus Paris Based on Steroidal Saponins. *Biochem. Syst. Ecol.* **2013**, 48, 163–173.
42. Zhao, Y.; Kang, L.-P.; Liu, Y.-X.; Zhao, Y.; Xiong, C.-Q.; Ma, B.-P.; Dong, F.-T. Three New Steroidal Saponins from the Rhizome of Paris polyphylla. Magnetic resonance in chemistry: MRC 2007, 45, 739–744.
43. Ur Rahman, S.; Ismail, M.; Khurram, M.; Ullah, I.; Rabbi, F.; Iriti, M. Bioactive Steroids and Saponins of the Genus Trillium. *Molecules*
44. Yang, Q.-X.; Xu, M.; Zhang, Y.-J.; Li, H.; Yang, C. Steroidal Saponins from *Disporopsis pernyi*. *Helv. Chim. Acta* **2004**, 87, 1248–1253.
45. Thu, Z.M.; Oo, S.M.; Nwe, T.M.; Aung, H.T.; Armijos, C.; Hussain, F.H.S.; Vidari, G. Structures and Bioactivities of Steroidal Saponins Isolated from the Genera *Dracaena* and *Sansevieria*. *Molecules* **2021**; 26; 1916.
46. Tran, Q.L.; Tezuka, Y.; Banskota, A.H.; Tran, Q.K.; Saiki, I.; Kadota, S. New Spirostanol Steroids and Steroidal Saponins from Roots and Rhizomes of *Dracaena angustifolia* and Their Antiproliferative Activity. *J. Nat. Prod.* **2001**, 64, 1127–1132.
47. Zhang, H.; Chen, L.; Kou, J.-P.; Zhu, D.-N.; Qi, J.; Yu, B.-Y. Steroidal Sapogenins and Glycosides from the Fibrous Roots of *Polygonatum odoratum* with Inhibitory Effect on Tissue Factor (TF) Procoagulant Activity. *Steroids* **2014**, 89, 1–10.
48. Yang, Y.; Jin, H.; Zhang, J.; Wang, Y. Determination of Total Steroid Saponins in Different Species of *Paris* Using Ftir Combined with Chemometrics. *J. AOAC Int.* **2018**, 101, 732–738.

49. Watanabe, K.; Mimaki, Y.; Sakagami, H.; Sashida, Y. Bufadienolide and Spirostanol Glycosides from the Rhizomes of Helleborusorientalis. *J. Nat. Prod.* **2003**, 66, 236–241.
50. Duckstein, S.M.; Lorenz, P.; Conrad, J.; Stintzing, F.C. Tandem Mass Spectrometric Characterization of Acetylated Polyhydroxy Hellebosaponins, the Principal Steroid Saponins in *Helleborus niger* L. Roots (#). *Rapid commun. Mass spectrom. RCM* **2014**, 28, 1801–1812.
51. Tian, L.W.; Zhang, Z.; Long, H.L.; Zhang, Y.J. Steroidal Saponins from the Genus *Smilax* and Their Biological Activities. *Nat. Prod. Bioprospect.* **2017**, 7, 283–298.
52. Wu, X.-H.; Wang, C.-Z.; Wang, S.-Q.; Mi, C.; He, Y.; Zhang, J.; Zhang, Y.-W.; Anderson, S.; Yuan, C.-S. Anti-Hyperuricemia Effects of Allopurinol Are Improved by *Smilax riparia*, a Traditional Chinese Herbal Medicine. *J. Ethnopharmacol*. **2015**, 162, 362 – 368.
53. Challinor, V.L.; Parsons, P.G.; Chap, S.; White, E.F.; Blanchfield, J.T.; Lehmann, R.P.; De Voss, J.J. Steroidal Saponins from the Roots of *Smilax* Sp.: Structure and Bioactivity. *Steroids* **2012**, 77, 504–511.
54. Mimaki, Y.; Watanabe, K.; Ando, Y.; Sakuma, C.; Sashida, Y.; Furuya, S.; Sakagami, H. Flavonol Glycosides and Steroidal Saponins from the Leaves of *Cestrum nocturnum* and Their Cytotoxicity. *J. Nat. Prod. 2001*, **64**, 17–22.
55. Yoshimitsu, H.; Nishida, M.; Nohara, T. Steroidal Glycosides from the Fruits of *Solanum abutiloides*. *Phytochemistry* **2003**, 64, 1361–1366.
56. Honbu, T.; Ikeda, T.; Zhu, X.-H.; Yoshihara, O.; Okawa, M.; Nafady, A.M.; Nohara, T. New Steroidal Glycosides from the Fruits of *Solanum anguivi*. *J. Nat. Prod*. **2002**, 65, 1918–1920.
57. Chen, L.; Yang, H.; Fang, Y.; Guo, W.; Chen, H.; Zhang, X.; Dai, W.; Chen, S.; Hao, Q.; Yuan, S.; et al. Overexpression of *GmMYB14* Improves High-Density Yield and Drought Tolerance of Soybean through Regulating Plant Architecture Mediated by the Brassinosteroid Pathway. *Plant Biochenol. J.* **2021**, 1, 702–716.
58. Lee, C.-L.; Hwang, T.-L.; He, W.-J.; Tsai, Y.-H.; Yen, C.-T.; Yen, H.-F.; Chen, C.-J.; Chang, W.-Y.; Wu, Y.-C. Anti-neutrophilic inflammatory steroidal glycosides from *Solanum torvum*. *Phytochemistry* **2013**, 95, 315–321.
59. Yokosuka, A.; Mimaki, Y.; Sashida, Y. Steroidal and Pregnane Glycosides from the Rhizomes of *Tacca chantrieri*. *J. Nat. Prod*. **2002**, 65, 1293–1298.
60. Kang, L.P.; Wu, K.L.; Yu, H.S.; Pang, X.; Liu, J.; Han, L.F.; Zhang, J.; Zhao, Y.; Xiong, C.Q.; Song, X.B.; et al. Steroidal saponins from *Tribulus terrestris*. *Phytochemistry* **2014**, 107, 182–189.
